# Superresolving the kidney—a practical comparison of fluorescence nanoscopy of the glomerular filtration barrier

**DOI:** 10.1007/s00216-020-03084-8

**Published:** 2020-12-05

**Authors:** Lucia C. S. Wunderlich, Florian Ströhl, Stefan Ströhl, Oliver Vanderpoorten, Luca Mascheroni, Clemens F. Kaminski

**Affiliations:** 1 Department of Chemical Engineering and Biotechnology, University of Cambridge, Cambridge, CB3 0AS UK; 2 Department of Physics and Technology, UiT The Arctic University of Norway, 9037 Tromsø, Norway; 3 Department of Nephrology, University Hospital Regensburg, 93053 Regensburg, Germany

**Keywords:** Kidney, Nephrin, Podocyte foot processes, Super-resolution microscopy, Fluorescence microscopy, Formalin-fixed paraffin-embedded

## Abstract

Immunofluorescence microscopy is routinely used in the diagnosis of and research on renal impairments. However, this highly specific technique is restricted in its maximum resolution to about 250 nm in the lateral and 700 nm in the axial directions and thus not sufficient to investigate the fine subcellular structure of the kidney’s glomerular filtration barrier. In contrast, electron microscopy offers high resolution, but this comes at the cost of poor preservation of immunogenic epitopes and antibody penetration alongside a low throughput. Many of these drawbacks were overcome with the advent of super-resolution microscopy methods. So far, four different super-resolution approaches have been used to study the kidney: single-molecule localization microscopy (SMLM), stimulated emission depletion (STED) microscopy, structured illumination microscopy (SIM), and expansion microscopy (ExM), however, using different preservation methods and widely varying labelling strategies. In this work, all four methods were applied and critically compared on kidney slices obtained from samples treated with the most commonly used preservation technique: fixation by formalin and embedding in paraffin (FFPE). Strengths and weaknesses, as well as the practicalities of each method, are discussed to enable users of super-resolution microscopy in renal research make an informed decision on the best choice of technique. The methods discussed enable the efficient investigation of biopsies stored in kidney banks around the world.

**Figure:**
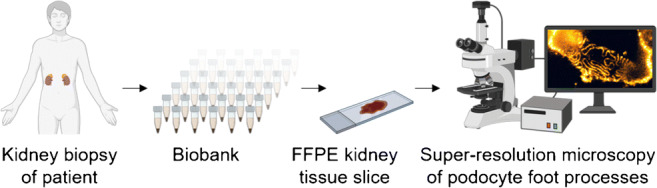
Graphical abstract

Graphical abstract

## Introduction

**Fig. 1:**
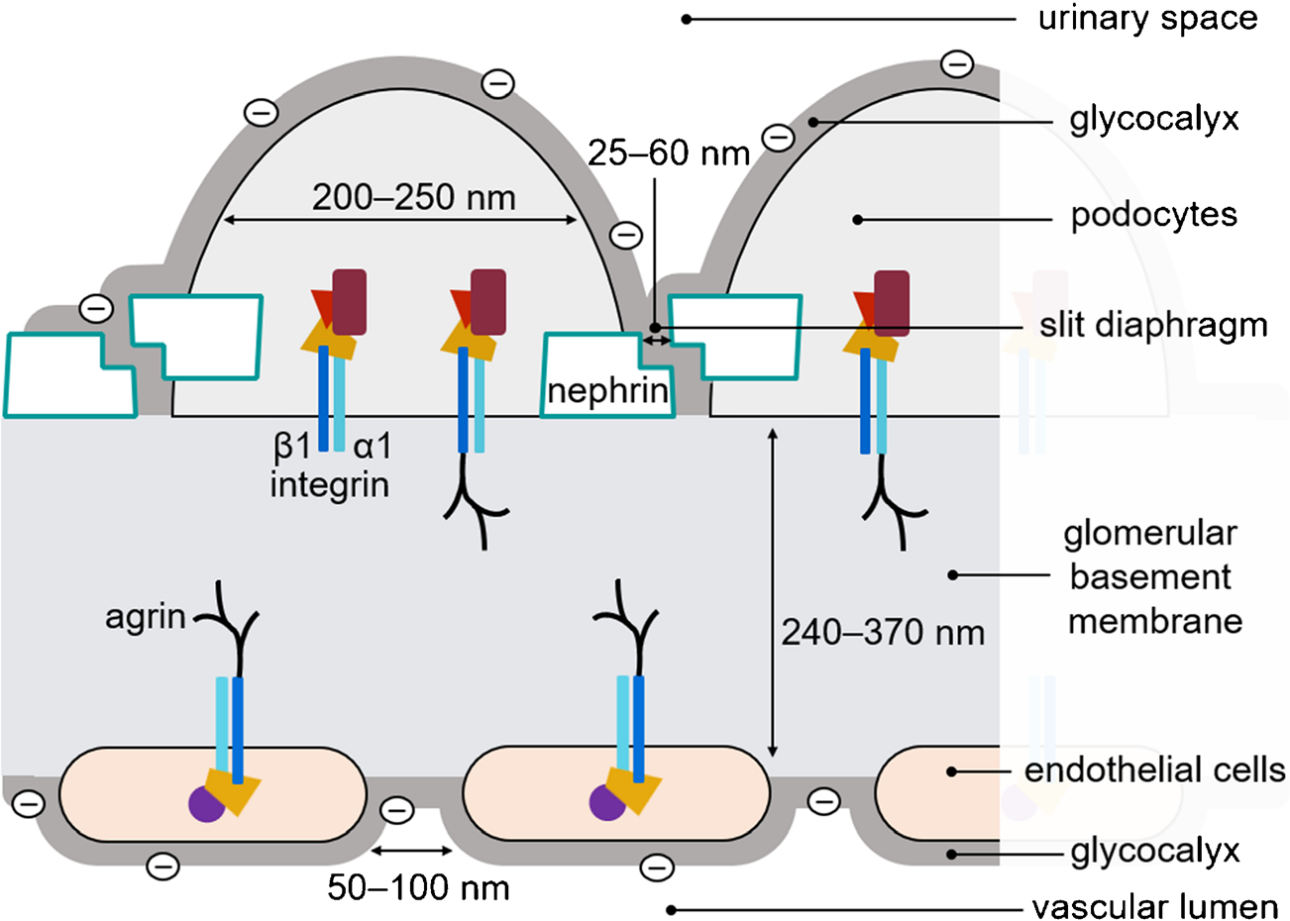
Schematic depiction of the glomerular filtration barrier

The glomerular filtration barrier (GFB) resembles a molecular sieve and is one of the main components of the renal corpuscle [1]. A disturbance of its fine architecture eventually leads to proteinuria, which represents an independent risk factor for progression of chronic renal disease as well as cardiovascular events like strokes or heart attacks [2]. The GFB separates blood from the urinary space and consists of three layers—the fenestrated endothelial cells, the glomerular basement membrane (GBM), and the epithelial podocytes (Fig. 1) [1]. An intact GFB prevents proteinuria [3], the urinary loss of macromolecules like albumin or immunoglobulins. Inside the vascular lumen, blood is filtered through various structures. First, it passes the glycocalyx of the endothelium [3], a coat of heavily branched and negatively charged polysaccharides, which are covalently attached to membrane proteins and lipids and are believed to repel negatively charged proteins [3]. This barrier is followed by the GBM, which is made up of a fine meshwork of proteoglycans, collagens, and anchoring proteins like integrin and agrin [4]. The final layer of the GFB is shaped by the foot processes of specialised epithelial cells, the so-called podocytes [5]. Similar to the endothelial cells, the podocytes are also covered by a glycocalyx [6]. Two neighbouring podocyte foot processes (PFPs) form the finest glomerular structure, the slit diaphragm, which measures about 40 nm in size [7]. When intact, the slit diaphragm prohibits the passage of macromolecules and their consecutive loss into the urine [5]. If altered, proteinuria occurs [8–12]. This happens, e.g. in hereditary mutations of nephrin or podocin, or under conditions of chronic kidney disease as caused by arterial hypertension or diabetes. Mutations of the *NPHS1* gene encoding for nephrin lead to congenital nephrotic syndrome of the Finnish type (CNF). In this disease, massive proteinuria is already present at the foetal stage. Affected children are born with a nephrotic syndrome including generalized oedema mainly due to the loss of albumin and an impaired immune system due to the loss of soluble components of the immune system like immunoglobulins. If not treated with kidney transplantation, the children usually die within their first 2 years of life [8]. Due to its striking role concerning the integrity of the filtration barrier and due to its sophisticated architecture [7, 8], the structural protein nephrin was chosen as the main object of comparison. Nephrin is a transmembrane protein that interacts homophilically with nephrin molecules of neighbouring PFPs and thus forms the major component of the slit diaphragm [13, 14]. Conventional immunofluorescence and electron microscopy (EM) are common tools to examine the structure of the GFB [15–17]. The latter provides a resolution down to the nanometre range, and thus even minimal changes of the barrier can be revealed. However, specific labelling with antibodies is limited due to the preparation protocol required for EM. As very thin sections of a few tens to hundreds of nanometres are required, biological samples first need to be fixed using, for example, glutaraldehyde and osmium tetroxide and embedded in certain acrylic resins. Alternatively, cryofixation or high-pressure freezing is used. These techniques solidify the specimens so that they can be cut to the desired thickness but at the same time hamper antibody penetration and, depending on the protocol, alter epitopes of proteins of interest by denaturation [18]. Moreover, the setup and its maintenance are expensive and experienced personnel is required to reach an acceptable throughput [19]. Conventional immunofluorescence microscopy, on the other hand, allows the labelling and detection of proteins of interest with unparalleled specificity and sensitivity, albeit at a relatively modest resolution. The latter is about 250 nm in the lateral direction due to optical diffraction as already recognised by Ernst Abbe around 1870 [20, 21]. With super-resolution microscopy, this diffraction limit is now readily overcome [22]. A number of approaches are in use, four of which in the investigation of renal tissue: single-molecule localization microscopy (SMLM) [23, 24]; stimulated emission depletion (STED) microscopy [25]; structured illumination microscopy (SIM) [26–28]; and expansion microscopy (ExM) [29–31]. The principles of each method are briefly introduced, and respective benefits and disadvantages are outlined in the Methods section.

Independently of the microscopy technique to be used, samples need to be correctly prepared prior to imaging them. Pathologists have conventionally resorted to elaborate sample treatments, i.e. cryosectioning or embedding in paraffin. Although frozen tissue samples are easier to prepare than formalin-fixed and paraffin-embedded (FFPE) samples, biopsies need to be frozen as soon as possible after extraction and require continuous storage at − 80 °C and thus are more vulnerable to technical failures. Frozen samples benefit from the preservation of RNA, DNA, and posttranslational protein modifications, which can be probed with techniques such as polymerase chain reaction, next-generation sequencing, mass spectrometry, and western blots. However, the formation of ice crystals can affect subcellular structures and introduce artefacts [32]. FFPE favours the preservation of tissue morphology and is therefore ideally suited for resolving fine structures, but it bears the risk of epitope masking due to overfixation. Despite these caveats, FFPE has become the most commonly deployed preservation method, because of the easy subsequent handling of samples at room temperature (RT), and facile storage over long time periods, often decades. Healthy and diseased human FFPE kidney biopsies have been imaged successfully with super-resolution microscopy [24, 27, 28] including a direct structural comparison of human and mouse podocyte foot processes. This report describes a significant higher density of the slit diaphragm (describing the slit diaphragm length per area of the glomerular capillary) in mice compared to humans [28]. However, the strategy for sample preparation of both species is the same, with a minor modification in the protocol for ExM of human tissue elaborated by Chozinski et al.

In conclusion, the application of super-resolution microscopy to conventionally prepared tissue sections in daily clinical routine opens the door to accelerating and improving diagnostics as well as tapping into the potential of biobanks all around the world for therapeutic research. This article provides a comprehensive comparison of the application of the super-resolution methods SMLM, STED, SIM, and ExM to FFPE slices of murine kidneys. Strengths and weaknesses as well as the practicalities of each technique will be discussed. The aim is to aid groups that lack previous experience with super-resolution microscopy techniques to make informed decisions on which method to choose for their specific question.

## Material and methods

### Preparation of formalin-fixed and paraffin-embedded mouse kidney slices

Wild-type C57BL6/N mice (Charles River) were anaesthetized with 12 mg/kg xylazine and 80 mg/kg ketamine followed by perfusion with PBS and 4% paraformaldehyde (PFA) for 5 min for fixation. Kidney perfusion was performed according to the *Guide for the Care and Use of Laboratory Animals* published by the US National Institutes of Health and was approved by the government of Lower Franconia. Subsequently, both kidneys were removed and placed in 70% methanol for several hours. For paraffin embedment, kidneys were immersed in methanol with increasing concentrations of 70% for 30 min, and 80%, 90%, and 100% each twice for 30 min. Samples were dipped in 100% isopropanol for 30 min at room temperature (RT) followed by 30 min at 45 °C, before being dipped in a mixture of isopropanol and paraffin at 60 °C for 30–60 min. They were immersed in pure paraffin for several days before embedding them in cassettes filled with paraffin at ambient temperature for storage. The PFFE kidneys were sliced into sections of 2 μm using a microtome (Leica Microsystems). Tissue sections were placed in a water bath heated to 42 °C to smooth out followed by picking them up with no. 1.5 precision cover glasses. Sections were dried at 42 °C on a flattening table before being stored at RT.

The FFPE kidney on no. 1.5 precision cover glasses were deparaffinised using xylene twice for 10 min and 2-propanol with decreasing concentrations of 100%, 96%, 80%, and 70% for 5 min each followed by dipping in phosphate-buffered saline (PBS). For antigen retrieval, samples were immersed in citrate buffer (1 mM citric acid, 0.05% Tween-20 in DDI water, pH 6) at 96 °C for 30 min. After cooling down to RT, they were submerged in PBS briefly before draining and encircling single kidney sections with a wax pen to keep fluids positioned on the tissue.

### Reducing autofluorescence of FFPE kidney tissue

For reducing autofluorescence of kidney tissue, we investigated several options: samples were incubated with either 0.2% sodium borohydride (NaBH_4_) or 100 mM glycine in PBS thrice for 10 min or 0.05% trypan blue (TB) in PBS for 15 min. Tissue was washed with PBS before immunolabelling. Alternatively, samples in PBS were put under a 430 mW white light LED with a distance of 10 cm between the sample and the light source for approximately 15 h. Different treated tissue sections that were compared regarding autofluorescence were illuminated with equal laser powers. For intensity analysis, the image field was centred on a glomerulus.

### Immunostaining of murine kidney slices

Afterwards, unspecific binding sites were blocked with blocking solution (10% donkey serum, 0.9% BSA, 0.05% Tween-20 in PBS) for 1–3 h and samples were labelled with polyclonal goat anti-nephrin antibodies (AF3159, R&D Systems) at 4 °C overnight. The next step was technique-dependent: for SMLM, anti-nephrin antibodies were diluted 1:500 in blocking solution to ensure sparsely blinking of fluorophores. For SIM and STED, primary antibodies were diluted 1:50 and for ExM 1:20 in blocking solution to guarantee dense enough labelling after expansion. Samples were washed with 1% BSA in PBS before detecting primary antibodies with Alexa Fluor 488-conjugated donkey anti-goat IgG (A-11055, Thermo Fisher Scientific) or Atto647N-conjugated rabbit anti-goat IgG (605456013, Rockland) at RT for 90 min. Secondary antibodies were diluted 1:2000 for dSTORM, 1:600 for SIM and STED, and 1:100 for ExM in blocking solution. After washing sections extensively, for ExM, nuclei were stained with Hoechst (1:600, 33342, Thermo Fisher, Scientific) for 20 min at RT and washed with PBS. Antibodies were postfixed with 2% PFA in PBS for 10 min before washing the tissue with PBS. Samples were handled under the absence of light after adding secondary antibodies.

### Single-molecule localization microscopy

In SMLM, images are acquired of individual molecules sequentially and computational fitting of a model point spread function (PSF) permits one to infer the position of the molecule at nanometre precision. The localization precision is thus not limited by diffraction, but only by the achievable signal to noise ratios (SNR). In direct stochastic optical reconstruction microscopy (dSTORM), one of the most widely used variants of SMLM, fluorophores are treated with a specific buffer, which enables their transition into a non-fluorescent state. This state is long-lived and inhibits emission of photons, thus maintaining the fluorophore in an ‘off’ state. At random, a subset of fluorophores can re-enter the normal ‘on’ state and emit photons. If this subset is sparse enough, the discrimination and subsequent localization of individual fluorophores are possible. This process of alternating subsets of ‘on’ fluorophore ensembles manifests itself by a characteristic ‘blinking’ during sample imaging and the actual position of a fluorophore is inferred from the centre of its detected PSF. Thus, the theoretical limit concerning resolution is determined by the accuracy of the fit as well as the proximity of the fluorophores to the protein they are linked to. This distance depends mostly on the method used for labelling, and ranges from ∼ 20 nm (indirect immunofluorescence) to∼ 10 nm (primary labelling), down to just ∼ 5 nm (Fab fragments) [33]. Like in a pointillist painting, sufficiently dense sampling of the underlying structure is required to reconstruct the distribution of fluorophores in the sample with high fidelity. In practice, this means that tens of thousands of images need to be acquired. The acquisition itself is hence time-consuming with respect to other super-resolution methods and furthermore frequently hampered by photobleaching or loss of blinking, which results in poor localization precision or in reconstruction artefacts. Moreover, labelling needs to be highly specific and dense to obtain a reliable representation of the underlying sample structure, which disqualifies a number of primary antibodies. Furthermore, the reconstruction algorithms are found to be prone to background signal and out-of-focus light, so images typically need to be taken in total internal reflection fluorescence (TIRF) instead of epifluorescence mode [22, 34].

The following protocol was used for SMLM: immediately prior to imaging, an object slide containing a cavity was filled with dSTORM buffer and placed on the coverslip carrying the sample. dSTORM buffer contains 70 mM mercaptoethylamine (MEA) and an oxygen scavenger system at a pH of 7.5. A 181 μl glucose stock solution (5 g glucose, 5 ml glycerine, 45 ml DDI water), 23 μl enzyme stock solution (100 μl catalase, 50 mg glucose oxidase, 25 ml glycerine, 200 μl TCEP (1 M), 1.25 ml KCl (1 M), 1 ml Tris-HCl (1 M, pH 7.5), 22.5 ml DDI water), 35 μl MEA stock solution (1.136 g MEA-HCl, 10 ml DDI water), and 4 μl KOH were added to 261 μl PBS. Samples were sealed with nail polish along the edges of the cover glass to avoid the formation of air bubbles. Imaging was carried out on a dSTORM microscope that was custom-built around an inverted microscope frame (IX-73, Olympus) and presented elsewhere in greater detail [35]. In short, fluorescent dyes were excited with a 647nm diode laser (VFL-P-300-647-OEM1-B1, MPB Communications Inc.), a 561 nm laser (Jive 500, Cobolt), a 488 nm laser (Sapphire, Coherent), and a 405 nm (LBX-405, Oxxius) diode laser for photo switching. For dSTORM, a 100×/1.49 NA oil immersion objective (UAPON100XOTIRF, Olympus) was used and images were relayed with a 1.3× magnification image splitter (TwinCam, CAIRN) and captured with an EM-CCD camera (iXon Ultra 897, Andor) using the camera software Solis (Andor). For each field of view, a time series of 20,000 to 50,000 frames with an exposure time of 5.6 ms was acquired. Samples were illuminated in TIRF mode with a laser power of 300 mW. For photo switching, a UV laser power of 1 to 2 mW was used. Widefield overview images were acquired with a 20×/0.7 NA air objective (UCPlanFL N, Olympus) using epifluorescence and low laser power prior to dSTORM imaging. The imaged area of the sample covered 256 × 256 camera pixels corresponding to an area of 41 × 41 μm^2^ of the sample. Reconstruction of dSTORM images was performed with the software rapidSTORM 3.3 [36] and images were analysed and processed with Fiji (NIH).

### Stimulated emission depletion microscopy

Like SMLM, STED microscopy theoretically grants unlimited resolution. Just as confocal microscopy, STED is a point scanning technique and produces an image by probing every pixel in the sample sequentially, albeit with a much smaller effective ‘probe area’. In a STED setup, an excitation beam matching the excitation maximum of the interrogated fluorophore is surrounded by a vortex beam used to induce stimulated depletion in the far-red end of the emission spectrum of the same fluorophore. Due to its ‘doughnut’ shape, the depletion beam de-excites fluorophores in a ring around the excitation beam to produce stimulated emission within a very small spectral window that can easily be filtered out. Hence, only fluorophores within the centre of the doughnut beam contribute effectively to the fluorescence signal. Increasing the intensity of the depletion beam shrinks the effective intensity minimum in the centre of the doughnut beam, thus improving the effective resolution. Although image acquisition in STED is much quicker than in SMLM, its point scanning characteristic still requires considerably more time to generate a single image than SIM or ExM do. Depending on the specimen, optical clearing protocols might be needed to achieve optimal image quality. Moreover, due to the high depletion intensities used, the sample is usually affected by photobleaching in the imaged area, which limits the use of multiple fluorophores. An advantage, on the other hand, is that STED is a purely optical technique, so no computational post-processing is required [37], which avoids reconstruction artefacts.

For STED imaging in practice, stained tissue was incubated in 2,2′-thiodiethanol (TDE) mounting with increasing concentrations of 10%, 25%, 63%, and 95% for 30 min at RT each and finally embedded in a 95% TDE solution. Cover glasses containing the samples were sealed on an object slide using nail polish. STED imaging was performed on a custom-built microscope with a commercial microscope frame (IX83, Olympus) (details on the system can be found elsewhere [38–40]). In brief, a titanium:sapphire laser (Mai Tai HP, Spectra-Physics) was used to generate a 647 nm excitation beam and a 765 nm depletion beam, shaped into a vortex beam by a spatial light modulator (X10468-02, Hamamatsu). Images were taken by using a 100×/1.4 NA oil immersion objective lens (UPLSAPO100XO, Olympus) and galvanometer mirrors (Quad scanner, Abberior Instruments) for raster scanning. An avalanche photodiode (SPCM-AQRH, Excelitas Technologies) detects signals, which are acquired with the software Imspector (Abberior Instruments). The field of view covered 1600 × 1600 camera pixels corresponding to an area of 80 × 80 μm^2^ of the sample. For image acquisition, a physical pinhole size of 2 mm, a pixel size of 20–35 nm, and a dwell time between 50 μs and 150 μs were used. Images were analysed and processed with Fiji.

### Structured illumination microscopy

SIM is often called an extended resolution technique [41] as its maximum resolution is about half the diffraction limit, and thus less than the other, true super-resolution methods. In SIM, the sample is illuminated with structured light patterns; most often, 9–15 sinusoidal stripe patterns are used [42], although image reconstructions with as few as three patterns have been demonstrated [43]. The stripes excite only fluorophores in the illumination maxima, thus allowing them to be discriminated from neighbouring fluorophores in the minima. To see and differentiate the non-illuminated fluorophores, the patterns need to be translated and rotated in a defined manner. The highest accessible spatial frequency (i.e. the finest detail in the sample) is given by the sum of the conventional widefield cut-off frequency (i.e. the inverse of the diffraction limit) and the spatial frequency of the illumination pattern modulation. As both widefield and pattern frequencies are diffraction limited, the maximal resolution is roughly twice the conventional resolution at best and thus, importantly, dependent on the excitation and emission wavelengths of the fluorophore. Shorter wavelengths provide higher resolution and hence, in multi-colour experiments, it is necessary to assign label colours such that each structure of interest is imaged with sufficiently high resolution. Under optimal conditions, a lateral resolution of ~ 100 nm can be achieved with the technique. Since the sample information is encoded in the raw data as frequency beats, it is necessary to extract higher resolution information computationally using dedicated reconstruction algorithms that are prone to artefacts. However, if these difficulties can be overcome and the maximum resolution is tailored to be sufficient for the task, SIM offers a high-throughput efficiency including the possibility to acquire large three-dimensional (3D) image stacks that are optically sectioned.

For SIM experiments, a small amount of PBS was added on the tissue and samples were covered with an object slide. Cover glasses were sealed with nail polish along the edges. SIM imaging was performed on a custom-built microscope (details can be found elsewhere [44]) based on a commercial microscope frame (IX-71, Olympus). A 488 nm laser (iBeam SMART, Toptica) was used for excitation and laser beams were shaped by using a ferroelectric spatial light modulator (Forth Dimension Displays M249) and a spatial mask made of aluminium containing six holes. A 60×/1.2 NA water objective lens (UPLSAPO60XW, Olympus) and a sCMOS camera (Hamamatsu Orca Flash v4.0) were used for detection and images were acquired with the software HCImage (Hamamatsu). The imaged area covers 1024 × 1024 camera pixels corresponding to an area of 42.65 × 42.65 μm^2^ on the sample. Raw data was processed into reconstructed SIM images applying a modified version of the Fiji plug-in FairSIM [45] (modified by Marcus Fantham, Laser Analytics Group). For reducing out-of-focus light, a combination of Richardson-Lucy deconvolution on the input image using a value of 0.05 and the Wiener filter on the output image using a value of 5 was applied.

### Expansion microscopy

ExM is the only super-resolution method that can be performed using conventional widefield or confocal light microscopes. This technique is based on physical magnification of the sample, i.e. the whole structure of interest is enlarged isotropically with a linear expansion factor of about 4.5×. Numerous ExM variations contain the following key steps of the original sample expansion protocol: immunostaining of the proteins of interest in the sample, linking of fluorophores to a polymer that crosslinks to form a hydrogel, digestion of surrounding tissue, and isotropic expansion of the hydrogel (with the fluorescent markers attached) through hydration. As proteins in the sample are digested and only linked fluorescent markers are left behind in a watery environment, this procedure also clears the sample from background fluorescence. The actual imaging is performed using conventional fluorescence microscopy and no specific computational post-processing is required. Furthermore, it is possible to combine expansion microscopy with a second super-resolution method, e.g. SIM, to further increase the resolution capacity [46]. ExM has been proven as a suitable method for performing multi-colour staining containing reference proteins inside the glomerular basement membrane such as agrin, which forms the basis of an application in diagnostics [31].

We used this protocol for ExM: tissue was immunostained as described earlier, before applying the protocol for tissue expansion published by Zhao et al. [29]. Briefly, 0.1 mg/ml 6-((acryloyl)amino) hexanoic acid (AcX) in PBS was added to the samples for 3 h at RT to enable proteins to be linked to the polymer. Slices were washed twice in PBS before incubation in monomer solution (2 M NaCl, 8.625% (w/w) sodium acrylate, 2.5% (w/w) acrylamide, 0.1% (w/w) N,N′-methylenebisacrylamide in PBS) for 20 min at RT. To initiate the gel-forming polymerization reaction, 0.2% ammonium persulfate (APS) was added to the monomer solution. Additionally, 0.01% 4-hydroxy-2,2,6,6-tetramethylpiperidine 1-oxyl (TEMPOL) and 0.2% tetramethylethylenediamine (TEMED) were added as gelling inhibitor and accelerator, respectively. TEMPOL, TEMED, and APS were stored as stock solutions of 0.5%, 10%, and 10% in DDI water, respectively, at − 20 °C. The gelling solution was placed on a piece of Parafilm and a coverslip containing the stained kidney slice was dipped upside down in the gelling solution. Microscope slides served as spacers. Gelation occurred at 37 °C for 90 min before gels were incubated in digestion buffer (50 mM Tris (pH 8), 25 mM EDTA, 0.5% Triton X-100, 0.8 M NaCl, 8 U/ml Proteinase K in DDI water) at 58 °C overnight. During digestion, the gel detached from the coverslip and was transferred into a dish after digestion was completed. Gels were washed in PBS and nuclei were stained with Hoechst (1:400, 33342, Thermo Fisher Scientific) for 20 min at RT. Finally, gels were expanded by placing them in DDI water for several hours and changing the water every 30 min. For imaging, gels were placed on glass-bottom dishes (ibidi GmbH), coated with 0.0004% poly-L-lysine, and imaged with a 20×/0.45 NA air (LUCPlANFL N, Olympus) or 60×/1.2 NA water (UPLSAPO60XW, Olympus) objective lens using a commercial fluorescent widefield microscope (IX83, Olympus) and the software Micro-Manager (Open Imaging). The wide field microscope uses high-power plasma light source (HPLS343, Thorlabs) in conjunction with FF01-434/17 and FF01-482/25 excitation filters and FF01-474/27 and FF03-525/50 emission filters (all Semrock). Alternatively, gels were imaged with a commercial fluorescent confocal scanning microscope (TCS SP5, Leica Microsystems) using a 63×/1.20 NA water objective (W CORR HCX PL APO, Leica Microsystems) and the software LAS AF (Leica Microsystems).

### Measuring protein distances

For quantifying the autofluorescence of the FFPE kidney tissue, the intensity of fluorescence signals was determined by measuring the mean grey value of the overall image with Fiji. Comparability of fluorescence intensities of different images was ensured by choosing similar areas of the kidney section with centring the microscope’s field of view on a glomerulus. The width of PFPs was determined by measuring the peak-to-peak distance of two neighbouring slit diaphragms, visualized by staining nephrin, at the centre of their visible length.

### Determination of the expansion factor

The expansion factor was determined by measurement of either nuclear (ExM^N^) or glomerular (ExM^G^) sizes pre- and post-expansion, respectively. For ExM^N^, average-sized nuclei within glomeruli were manually encircled using Fiji. Nuclear sizes were measured before and after expansion. The expansion factor is calculated by using the formula:

1 $$ EF=\sqrt{\frac{A_{\mathrm{nucleus}\mathrm{exp}}}{A_{\mathrm{nucleus}}}} $$

with *A*_nucleus_ being the mean area of several nuclei before and *A*_nucleusexp_ being the mean area of nuclei after expansion.

For ExM^G^, the mean of the diameter of multiple individual glomeruli imaged after expansion divided by their respective diameter before expansion was taken.

## Discussion and results

### Effect of background signal

**Fig. 2:**
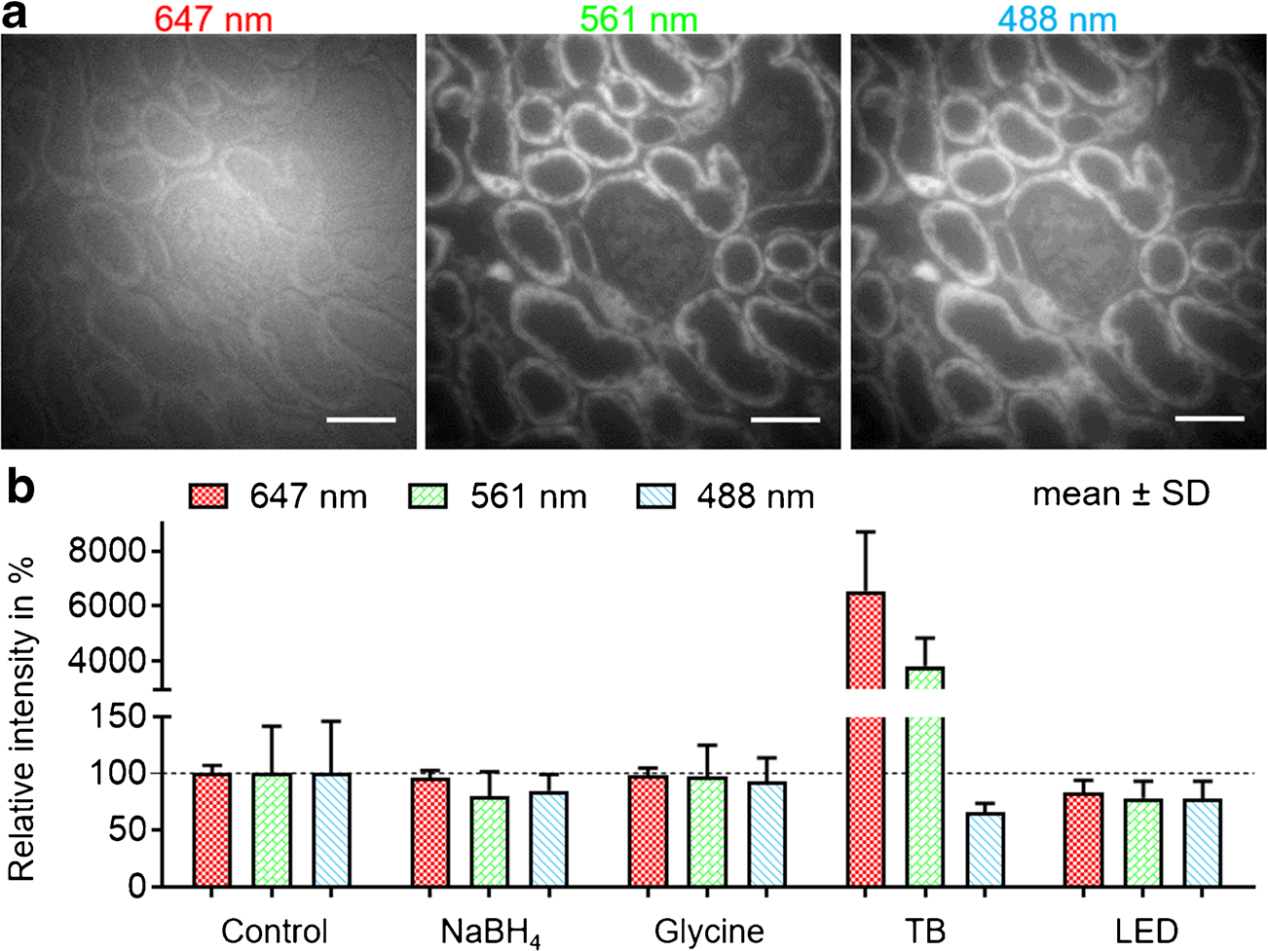
**a** Autofluorescence of unstained formalin-fixed, paraffin-embedded murine kidney slices upon excitation with wavelengths of 647 nm, 561 nm, and 488 nm, respectively. **b** Intensities of × 100 magnified areas inside glomeruli at administration of either NaBH_4_, glycine, trypan blue (TB) or LED. *n* ≥ 12

Super-resolution microscopy requires the use of vigorous control experiments to reduce artefacts that could be mistaken for true information. Unspecific background and autofluorescence are two of the most common sources of low-quality images. The autofluorescence of unstained FFPE murine kidney slices was determined upon illumination with laser light at excitation wavelengths of common fluorophores (Fig. 2). The fluorescence intensity for 488 nm and 561 nm peaked pronouncedly in the tubular system, while illumination at 647 nm resulted in unspecific, homogenous, and, as expected, overall lowest autofluorescence. NaBH_4_, glycine, bleaching with UV LED light, and trypan blue were applied as described in the methods section in an effort to reduce autofluorescence. As shown in Fig. 2, no significant improvement could be obtained with the first three substances. Although UV illumination has been reported to decrease the autofluorescence in other highly intrinsic fluorescent FFPE tissues, it does not have a great impact on kidney tissue despite higher illumination intensity than the one used in the present work [47]. Treatment with trypan blue, on the other hand, lowered the autofluorescence in the blue channel at the expense of a vast increase in the red and green channels, respectively.

In addition to the approaches used here, there are other common methods to reduce the autofluorescence for instance treatment with Sudan Black B, hydrogen peroxide, or ammonia [47, 48] or commercial kits such as TrueVIEW (Vector Laboratories, USA). However, certain tissue types including renal tissue contain an abundance of intrinsic highly fluorescent components such as NAD(P)H molecules, lipofuscin granules, red blood cells, and components of the extracellular matrix like flavins, collagen, and elastin [49]. These molecules have a major impact on the renal autofluorescence as kidney tissue is highly vascularized, shows a high metabolic rate, and is rich in lipofuscin. The deposition of lipofuscin is a general characteristic of ageing tissue and its accumulation is significantly increased in aged kidney biopsies [50]. With respect to applying super-resolution microscopy for diagnostic purposes, tissue samples are more likely to originate from elderly patients and thus autofluorescence might become a major issue. It has been observed in numerous studies on human [51, 52] as well as murine FFPE kidney tissue and its full reduction proved challenging [47, 53, 54]. Therefore, if a method other than SMLM is used, we suggest not to invest in reducing the background due to its negligible effect on kidney tissue autofluorescence. If the structure of interest is the meandering pattern of the slit diaphragm visualized with high quantum yield fluorophores, as done in this work, it might still be well distinguishable from the background even if its intensity is exceeding the one in the present study. In case the interpretation of structural characteristics becomes difficult due to high background signal, we find that performing ExM and making use of its beneficial effect of tissue clearing is the most practical option. As the ExM protocol is an extension of the conventional procedure of immunostaining, researchers can image their samples with standard immunofluorescence methods first and if the resulting data is inadequate because of high autofluorescence subsequently apply the ExM protocol to the same samples.

### Imaging performance of techniques

**Fig. 3:**
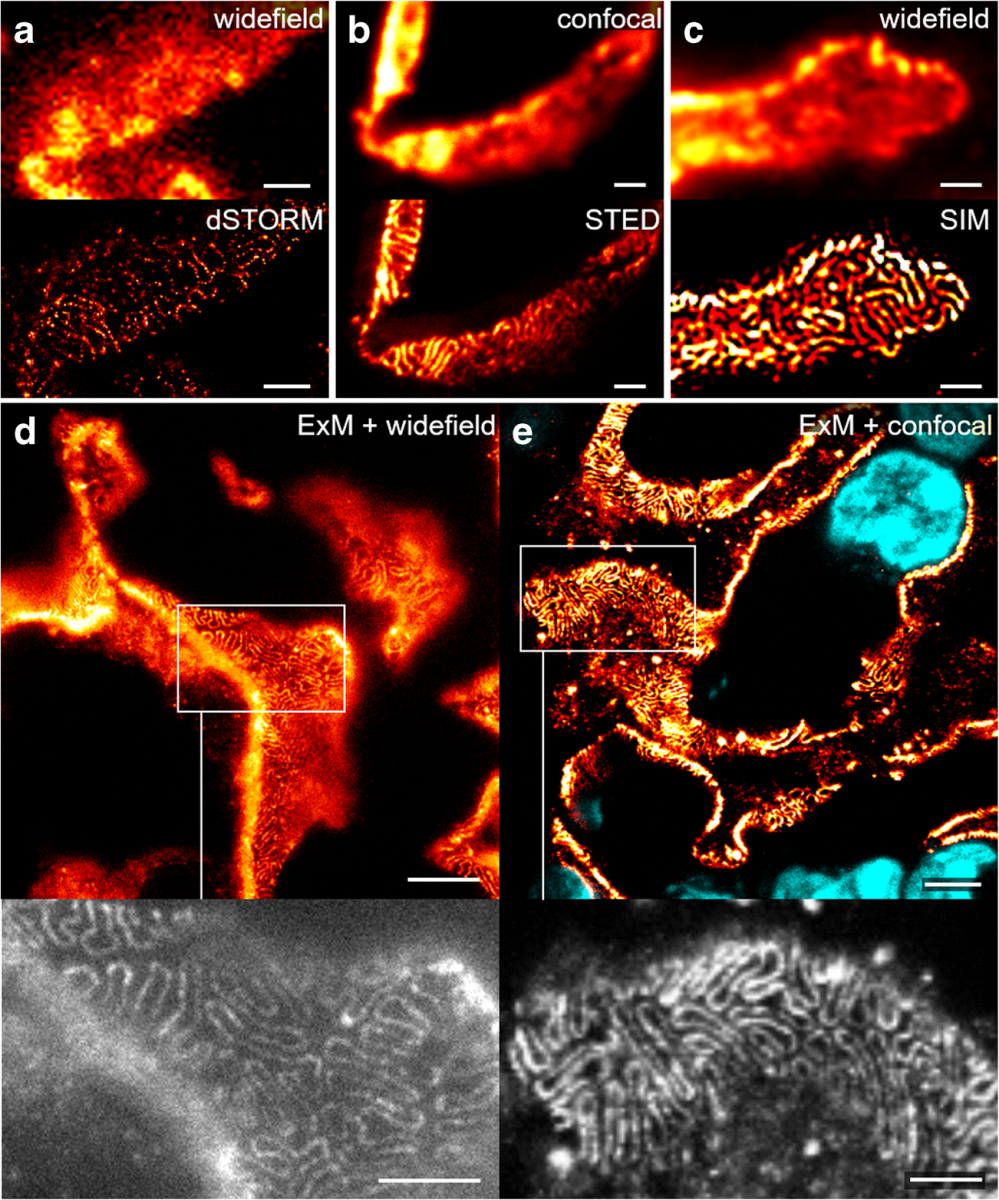
Comparison of nephrin imaging with different conventional and super-resolution modalities. **a** Widefield vs dSTORM using Atto647N. **b** Confocal vs STED using Atto647N. **c** Widefield vs SIM using Alexa Fluor 488. **d** ExM prepared sample imaged with widefield vs **e** confocal using Alexa Fluor 488. The confocal ExM image furthermore shows the nucleus stained with Hoechst. Highlighted panels show × 3 zoomed areas. Scale bars in **a**–**c** are 1 μm, in **d** and **e** 10 μm and in the zoomed in areas 5 μm (after expansion)

For the comparison of modalities, nephrin was chosen as the main protein to image for several reasons. First, it was subject of previous super-resolution studies on kidney slices [24, 25, 27, 30] and, second, due to the distinct pattern of the structures where the protein resides, it offers an ideal target for a comparative study. Visualization of nephrin showed a meandering pattern if viewed *en face* and a dotted line pattern along the convex side of a longitudinal section of the glomerular filtration barrier, which has been directly compared in correlative STORM-EM studies [24]. Example images obtained with the different techniques are shown in Fig. 3.

**Fig. 4:**
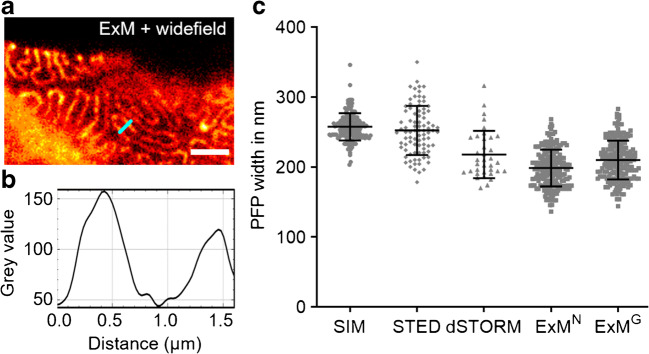
Podocyte foot process (PFP) width as determined by the different super-resolution techniques. **a** An ExM image serves as an example in order to show the peak-to-peak distance of neighbouring fluorescence intensity maxima **b** in areas of hairpin-like distribution of nephrin that was considered equivalent to the width of PFPs (blue line in **a**). Scale bar 5 μm (after expansion). **c** PFP width as determined by SIM (*n* = 159), STED (*n* = 87), dSTORM (*n* = 36), and ExM^N^/ExM^G^ (*n* = 210); expansion factor determined by nuclear and glomerular expansion, respectively. Bars represent mean ± SD

Regarding SMLM, single-molecule localization was only achieved efficiently in the far-red channel using Atto647N due to the high autofluorescence in the other channels. Furthermore, the pointillist images often showed marked gaps in the structure and hence proved difficult to evaluate reliably. Overall, the success rate was low due to background issues, which speaks against this technique for high-throughput studies. However, the localization precision heavily depends on the labelling strategy and labelling density [33] and thus varies between different targets. For nephrin imaging with STED microscopy, Atto647N was used as it is a robust dye providing good signal and depletion efficiency. In the resulting superresolved images, individual PFPs can be clearly distinguished and, in comparison to SMLM, without reconstruction artefacts. SIM imaging, on the other hand, was performed with Alexa Fluor 488, as the maximum resolution of SIM depends on the excitation wavelength of the utilized fluorophore. Using the blue channel, a resolution approaching 100 nm was achieved, which was sufficient to visualize the distinct meandering pattern of nephrin in the GBM. Finally, ExM was applied. An expansion factor between 4 and 5× enabled the clear resolution of PFPs with a conventional widefield or confocal microscope, respectively. However, as the expansion factor might vary slightly, a precise determination of the resolution proved challenging. Nevertheless, the observed distribution was a meandering pattern consistent with the findings acquired with the other methods presented above. Each of the four investigated super-resolution techniques was visually sufficient to resolve single PFPs; quantitatively, the foot process width was chosen as a metric. It was determined by measuring the peak-to-peak distance of the hairpin-like distributed nephrin when imaged *en face* (Fig. 4). The number of measured PFPs for SIM was 159 PFPs from 34 glomeruli of three kidney slices, for STED 87 PFPs from 16 glomeruli of four kidney slices, for dSTORM 36 PFPs from eight glomeruli of one kidney slice, and for ExM^N^/ExM^G^ 210 PFPs from 42 glomeruli of four kidney slices. For determining the expansion factor by nuclear expansion, 176 nuclei from 38 glomeruli of four kidney slices were measured before expansion and 156 nuclei from 39 glomeruli of three kidney slices after expansion. The expansion factor by glomerular expansion was determined by measuring 11 glomeruli from two kidney slices. The mean PFP width was 259 ± 19 nm (SIM), 252 ± 35 nm (STED), 217 ± 34 nm (dSTORM), 198 ± 26 nm (ExM^N^), and 210 ± 28 nm (ExM^G^), respectively. The variability between results obtained from different experiments using the same technique demonstrates their respective reliability. SIM and ExM showed the least standard deviation of ± 19 nm and ± 36 nm (ExM^N^)/± 38 nm (ExM^G^), respectively, followed by STED (± 35 nm) and SMLM (± 34 nm). The difference of the means of all applied techniques was considerable, so (I) cross-checking results with at least one other technique and (II) sticking to the same method for consecutive studies are heavily recommended.

### Ease of use of techniques

Samples were fixed by perfusion with paraformaldehyde. Note, however, that nanoscopy studies of cancer tissue [55] and placental tissue [56] found that features below 450 nm and 100 nm, respectively, can be lost in FFPE-preserved samples during fixation and thus are only feasible on, e.g. well preserved cryosections lacking morphologic aberrations due to the freezing process. Conventional immunostaining was the next core step regarding the sample preparation for each technique and takes 2 days. For SIM, this is already sufficient to proceed to the imaging. If the structure of interest requires a resolution of about 100 nm only, this is also true for STED. To reach the maximum resolution of STED, however, it is recommended to optically clear the tissue, which is a sophisticated and time-consuming procedure [25]. Additional preparation is also required for SMLM. First, the slices have to be cut as thin as possible to reduce background and out-of-focus light. For FFPE tissue, 2 μm was the thinnest thickness that could be cut routinely in this study. To narrow the gap between the objective and the sample as much as possible, the slices were put on coverslips instead of microscope slides. Slides containing a cavity opposed the sample to grant optimal coverage of the quenching buffer. This buffer needs to be optimised to any individual composition of fluorophores [34]. For ExM, the protocol for the actual expansion can be done subsequently to the staining only interrupted by the acquisition of widefield images for orientation and pre-expansion fluorescence images for determination of the expansion factor. The expansion extends the overall duration of the sample preparation by one day.

The actual imaging is straightforward for SIM and ExM, which also allows acquisition over very large fields of view or 3D stacks [27, 31]. Scanning larger areas with STED is more time-consuming but certainly practicable. However, the high laser intensities required for STED demand the use of very stable fluorophores to minimise the effects of bleaching; repetitive measurements of the same region of interest (ROI) and with the same wave were not possible in the setup reported here. Due to the necessity to image thousands of individual raw data images, the imaging process takes several minutes for SMLM regardless of the size of the area of interest. For SIM, STED, and dSTORM, dedicated imaging systems were used, whereas ExM can be applied on any less expensive conventional widefield or confocal microscope and is thus not restricted in the selection of fluorophore colours as long as they do not belong to the cyanine family of dyes such as Alexa Fluor 647 or Cy5, which are degraded during the polymerization step [57]. Moreover, the ease of use of ExM is fairly simple as, besides handling the gel, it does not require additional skills for microscope operation than for conventional fluorescence imaging. ExM and STED require no specific post-processing steps. However, for STED and SIM, a certain knowledge about the operating principle to adjust acquisition settings and image reconstruction, respectively, is necessary. SIM images are reconstructed in real-time. Due to artefacts, manual discrimination and selection of PFPs are essential, even though semi-automated evaluation methods have been published recently [27]. dSTORM requires the skill of preparing an ideal composed quenching buffer as well as the ability of reconstructing images. Its reconstruction takes between some minutes and a few hours depending on the algorithm and the size of the ROI. Moreover, several giga- or even terabytes may be required for storage of the unprocessed data.

Beyond these four imaging techniques, there are potential alternatives to achieve a resolution below 100 nm. A combination of ExM and STED, ExSTED, has been shown in extrarenal tissue [58]. Combining ExM and SIM resulted in an increase of artefacts (data not shown) without notable changes in the maximum resolution due to the reduced signal strength of expanded samples. Hence, this combination is not recommended for straightforward use in PPFE kidney tissue. This issue was overcome in the expansion of the Drosophila synaptonemal complex using an extended protocol spanning a period of 5 days [46]. Moreover, enhanced protocols for ExM such as iterative ExM (iExM) [59] and X10 ExM [60] improve the resolution over original protocols. They have not yet been published in renal tissue but in murine brain, liver, lung, and rat brain, respectively, and should theoretically be adoptable.

The discussed aspects for all applied techniques are summarized in Table 1.

**Table 1:** Comparison of SIM, STED, dSTORM, and ExM for imaging of formalin-fixed, paraffin-embedded renal tissue

	SIM	STED	dSTORM	ExM
Preparation time	2 days	2 days	2 days	3 days
Acquisition time	Milliseconds	Scanning technique, seconds	Seconds	Milliseconds to seconds (widefield/confocal)
Reconstruction time	Seconds	None	Seconds to minutes	None
PFP width (mean ± SD) *(published distance)*	259 ± 19 nm *(249 nm in human samples* [27]*)*	252 ± 35 nm *(220–260 nm* [25]*)*	217 ± 34 nm *(200–300 nm* [24]*)*	198 ± 26 nm/210 ± 28 nm *(247 ± 29 nm* [31]*)*
Caveats	Reconstruction artefacts show patterns similar to PFPs	z-Stacking difficult due to quick bleaching of fluorophores	Dotty representation of PFPs, thus lack of complete localization information	Expansion factor varies slightly between experiments; hence, it is challenging to determine exact absolute distances
Ease of use	Moderate	Moderate	Challenging	Simple
Costs	Dedicated system	Dedicated system	Dedicated system	Conventional system
Overall recommendation	++	+	–	+++

## Conclusion

Optical super-resolution microscopy enables the nanoscale imaging of specifically labelled samples and thus combines advantages of both light and electron microscopy. In this paper, four types of super-resolution microscopy techniques were compared regarding their applicability to FFPE renal tissue and their practicality: single-molecule localization microscopy (SMLM), stimulated emission depletion (STED) microscopy, structured illumination microscopy (SIM), and expansion microscopy (ExM). It was possible to resolve single PFPs with each of the techniques. Foot process width ranged from 198 to 259 nm in the same range as measurements previously reported [24, 27, 30, 31]. However, for superresolving FFPE tissue, there is no ‘one-fits-all’ technique. The method of choice depends heavily on the specific aim. If investigating structures that require a resolution less than about 100 nm, SIM or ExM are sufficient. A SIM setup is costly but does not need further consumable reagents for operation, offers high-throughput capacity, and does not damage the sample. Hence, this technique is recommended for imaging large or large numbers of samples. In the case of just occasional need for super-resolution microscopy, ExM will most likely give adequate results and benefits from the application to a conventional fluorescence or confocal microscope. For a resolution below 100 nm, using STED or SMLM is recommended. STED is the commercially most expensive technique but provides easy handling as well as fast and reliable results. Although SMLM can be applied to FFPE tissue, the slices that can reasonably be cut are still not thin enough to minimise background fluorescence. Thus, renal autofluorescence limits the number of potential fluorophores dramatically. If a SMLM setup is available and utilized for renal imaging, cryosectioning of tissue is recommended. Regardless of the imaging technique, it should be noted that the quality of preservation of histologic structures might be limited in case of immersion fixation, which is used for clinical samples, and thus has a growing impact with increasing resolution. Nevertheless, we see great potential for using super-resolution microscopy as a tool for therapeutic research as well as for diagnostic analysis of clinical biopsies of kidney patients.

## Data Availability

The datasets generated and analysed during the current study are available from the corresponding author on reasonable request.
